# Radiosensitizing effect of astaxanthin on radiation–induced cytotoxicity in breast cancer cells

**DOI:** 10.1186/s43046-026-00352-z

**Published:** 2026-06-08

**Authors:** Bothaina AL- Awady, Abdel-Moneim Osman, Diana Hanna, Shereen El kiki, Ebtehal El-Demerdash, Marwa Sharaky

**Affiliations:** 1 National Cancer Institute, Cancer Biology Dept., Pharmacology Unit, Cairo University, Giza, Egypt; 2 Faculty of Pharmacy, Dept. of Pharmacology & Toxicoloy, Ain Shams University, Cairo, Egypt; 3 National Centre for Radiation Research and Technology, Egyptian Atomic Energy Authority, Cairo, Egypt

**Keywords:** Radiation, Astaxanthin, Breast cancer cells, Radiosensitization

## Abstract

**Background:**

Radiotherapy (RT) is a primary treatment for cancer, including breast cancer, but its effectiveness is limited by severe side effects. Radiosensitization using natural products can enhance efficacy while reducing radiation doses. Astaxanthin (AST), a natural carotenoid with antioxidant properties, requires further investigation for its role in cancer therapy. Especially in many previous studies, AST found to has prooxidant effect in cancer cells reverse its antioxidant effect in normal cells.

**Methods:**

This study explored AST’s potential to enhance the cytotoxic effects of ionizing radiation (IRR) against MCF7 breast cancer cells and the underlying mechanisms. Cytotoxicity, apoptosis induction, anti-metastatic activity, and cell cycle distribution were analyzed in response to IRR with and without AST.

**Results:**

IRR alone showed limited cytotoxicity, whereas addition of AST significantly enhanced IRR’s effects, reducing IC_50_ by ten folds in combination treatment. AST increased apoptosis markers (caspase-3 and BAX/BCL2 ratio) by 16% and 67.8%, respectively. It also decreased proliferation index and S-phase ratio by 43.24% and 42.6%, respectively. AST further reduced metastatic markers MMP2 and MMP9 by 25% and 33.8%, respectively, in combination therapy. Additionally, AST exhibited a pro-oxidant effect, elevating MDA and H₂O₂ levels by 16.9% and 18.9%, respectively. It also caused cell cycle arrest at the S and G2/M phases, making cells more susceptible to IRR.

**Conclusion:**

These findings confirm AST’s radiosensitizing effect via apoptosis induction, cell cycle disruption, and anti-metastatic activity, supporting its potential in enhancing radiotherapy efficacy for breast cancer cells. Further research is warranted to explore its application in cancer treatment.

## Introduction

Many epidemiological studies proved that breast cancer is a prevalent reason of mortality among Women aged 35–55 years old [1, 2]. Different treatment strategies are now available for various breast malignancies, including surgery, chemotherapy, immunotherapies and radiotherapy. Radiotherapy (RT) is considered as a primary option for many types of cancer either alone or in combination with surgery and chemotherapy. It has been shown in clinical studies that more than half (about 70%) of breast cancer patients are exposed to RT. Moreover, in some cases RT is considered the only kind of breast cancer treatment [3]. In this type of treatment, high doses of radiation therapy are used to destroy cancer cells, which is restricted as it may cause serious side effects [4, 5]. Radiation therapy may cause fibrosis and inflammation along with apoptosis in many cases through DNA damage [6].

In an attempt to minimize anticancer effective doses of radiation and chemotherapy and thereby their side effects, a variety of approaches have been investigated. One of them is the addition of natural compounds with chemopreventive or anticancer properties to the anticancer treatment protocol [7]. Astaxanthin (AST) is a carotenoid dye that belongs to the xanthophyll’s family. The green microalgae *haematococcus pluvialis* is one of the natural sources of marine AST. AST is also synthesized for commercial uses as a dietary supplement [8]. It has a potent antioxidant, antitumor, anti-inflammatory, anti-lipid peroxidation and cardio protective effects [9, 10]. McCall et al. [11] found that application of AST significantly reduced proliferation rates and inhibited breast cancer cell migration compared to control normal breast epithelial cells. Moreover, Osman et al. [7] reported chemosensitizing effect of AST in breast cancer cells to the action of radiomimetic drug doxorubicin. While, there is a current gap about radiosenstization of AST with radiation therapy in breast cancer in vitro and in vivo.

Therefore, the current study was directed to evaluate the significance of employing one of the natural product namely astaxanthin to RT, aiming to increase the effectiveness of RT. Thereby, the RT dose could be decreased with little side effects. Moreover, this work aimed to investigate the possible underlying mechanisms of different pathways including apoptosis, metastasis and cell cycle phases distribution.

## Materials and methods

### Drugs and chemicals

AST was obtained from Haihang Industry Co., Ltd. (china). The stock solution was dissolved in dimethyl sulfoxide (DMSO) and preserved at −20^0^C. The solution was diluted in (RPMI) media or PBS immediately before each experiment to the desired final concentrations. All other chemicals and Kits were purchased from Elabscience Houston, Texas, 77,079, (USA) and biodiagonistic (Cairo, ARE).

### Irradiation

MCF7 cells were exposed to six doses of Ƴ-irradiation (2,4,6,8,10,12 Gy) at the National Centre for Radiation Research and Technology (NCRRT), Cairo, Egypt, using an AECL 137Cs Gamma Cell-40 biological irradiator.

### Cells and cell cultures

Human breast cancer cell line MCF7 was obtained from ATCC (American tissue culture collection) and maintained in Pharmacology Unit, National Cancer Institute, Cairo University, Egypt. The adherent cells were grown as a monolayer culture in RPMI supplemented with penicillin (100 IU/ml), streptomycin (100 μg/ml) and 10% fetal bovine serum.

Cells were cultured at 37^0^C in humidified 5% CO_2_ atmosphere and were passaged every 4–5 days.

### Assessment of cytotoxic activity

Cytotoxic activity of IRR and/or AST was determined using the Sulforhodamine (SRB) method as previously described by Skehan et al. [12]. In brief, cells were plated in 96 well microtiter plates at a concentration of 5 × 10^3^ cells/well with RPMI-supplemented medium. After 24 h incubation period, AST was added at different concentrations (75, 150, 225 μg/ml). Two hours later, cells were exposed to IRR with rate of (o.588cGy/sec.) to induce exposure of different Grays (2, 4, 6, 8, 10 and 12 Gy). Following incubation for 48 h, cells were fixed by adding 50 μl of cold 50% TCA for 1 h at 4°C. Supernatant was then discarded and the wells were washed with distilled water, air dried, stained for 30 min at room temperature with 0.4% SRB dissolved in 1% acetic acid and then washed with 1% acetic acid. The plates were then allowed to air dried, and the dye was dissolved in 100 μl/well of 10 mM Tris base (pH 10.5) for 10 min. Each well's optical density (OD) was measured spectrophotometrically at 490–530 nm using an ELISA microplate reader (TECAN SunriseTM, Germany), with automated shaking taking place for 30 s. before reading. The percentage of cell survival was calculated as follows:

$$\mathrm{Survival}\;\mathrm{fraction}=\mathrm{OD}\;\left(\mathrm{treated}\;\mathrm{cells}\right)/\mathrm{OD}\left(\mathrm{control}\;\mathrm{cells}\right)$$

The IC_50_ values (the doses of irradiation and/or AST required to produce 50% inhibition of cell growth) were calculated using sigmoidal dose response curve-fitting models (GraphPad, Prizm5 software incorporated).

In all the following experimental work, the IRR of dose 4Gy with or without approximately half IC_50_ of AST in MCF7 cell line was used to study mechanistic pathway of astaxanthin with IRR. Assessment of each parameter were carried out three independent times.

### Determination of malondialdehyde (MDA) and hydrogen peroxide (H_2_O_2_) content in MCF7 cells

Lipid peroxidation product (MDA) and Hydrogen Peroxide (H_2_O_2_) were quantified by measuring their levels in cell culture lysate of control and treated cells using MDA and H_2_O_2_ Assay Kits (Biodiagnistic, Cairo, ARE, Catalog No. MD 25 29 and HP 25, respectively) following the manufacturer’s instructions. The absorbance at 540 nm and 510 nm, respectively were measured spectrophotometrically following incubation period of 30 min using an ELISA microplate reader (TECAN SunriseTM, Germany). The MDA and H_2_O_2_ concentrations were calculated as follow:

$$\mathrm{MDA}=\left(\mathrm{A}_\mathrm{Sample}\;/\;\mathrm{A}_\mathrm{Standard}\right)\times 10/ \mathrm{gm}\;\mathrm{protein}$$

$$\mathrm{H}_\mathrm{2}\mathrm{O}_\mathrm{2}=\{\left(\mathrm{A}_\mathrm{Sample}/\mathrm{A}_\mathrm{Standard} \right)\times 0.5\}/\mathrm{gm}\;\mathrm{protein}$$

### ELISA assay

#### Determination of caspases 3 and 9 concentrations in MCF7 cell lysate

The apoptotic activity was determined by measuring the protein concentrations of caspases 3 and 9 spectrophotometrically at 450 nm in cell lysate using ELISA Assay Kits (Elabscience® Human ELISA Kit, Houston, Texas, 77,079, USA, Catalog No. E-EL-H0017 and E-EL-H0663, respectively) following the manufacturer’s instructions. Percentage changes in caspase 3 and 9 concentrations were determined by comparing the absorbance of treated cells with the untreated control.

#### Determination of (BAX and BCL2) concentrations in MCF7 cell lysate

BAX and BCL2 concentrations were measured spectrophotometrically at 450 nm in treated and control cell lysate using ELISA Assay Kits (Elabscience® Human ELISA Kit, Houston, Texas, 77,079, USA, Catalog No. E-EL-H0562 and E-EL-H0114), respectively following the manufacturer’s instructions.

#### Determination of MMP2 and MMP9 concentration in MCF7 cells

The inhibition of metastatic activity was measured by quantifying the levels of MMP2 and MMP9 enzymes spectrophotometrically at 450 nm in cell lysate using ELISA Assay Kits (Elabscience® Human ELISA Kit, Houston, Texas, 77,079, USA, Catalog No. E-EL-H6075 and E-EL-H0114, respectively) following the manufacturer’s instructions. MMP2 and MMP9 concentrations were determined by comparing the absorbance of treated cells with the untreated control.

#### Determination of PARP-1 and MMR concentrations in MCF7 cell lysate

The DNA repair activity was determined by measuring the concentrations of PARP1 and MMR spectrophotometrically at 450 nm in treated and control cell lysate using PARP1 ELISA Assay Kit (Abcam company Cambridge, UK, Catalog No. ab285289) and RayBio® MMR ELISA Kit (Guangzhou, Guangdong, China, Catalog No. ELH-MMR), respectively following the manufacturer’s instructions.

#### Determination of (CDK4 and CDK6) concentrations in MCF7 cell lysate

Concentrations of CDK4 and CDK6 were measured spectrophotometrically at 450 nm in cell lysate using ELISA Assay Kits (MyBioSource, San Diego, USA, Catalog No. MBS7004 and MBS2020199, respectively) following the manufacturer’s instructions. CDK4 and CDK6 concentrations were determined by comparing the absorbance of treated cells with the level of the untreated control.

#### Cell cycle analysis using flow cytometry

Cells were plated in 12-well plates at a cell density of 6–8 × 10^5^ cells/well in RPMI supplemented medium. Twenty-four hours later, cells were exposed to AST (75 µg/ml) and/or IRR (4Gy) with rate of 0.588cGy/sec. After incubation for 48 h. the cell medium was then removed and washed once with PBS. Cell cycle analysis was performed according to the method of Pozarowski and Darzynkiewicz [13], using a flow cytometer (Becton Dicknoson (BD) FACSCalbur, USA).

#### Determination of protein concentration

After incubation of untreated and treated cells for the specified times, media were collected and stored at − 80 °C. Cells were harvested by trypsinization then lysed with RIPA lysis buffer (25 mM Tris HCL pH 7.6, 150 mM NaCl, 1% triton X-100, 1% sodium deoxycholate and 0.1% SDS) containing protease inhibitors. Protein concentrations in media and cell lysate were determined by Bradford Assay kit (Pierce, Rockford, IL). All experiments were run in triplicate.

#### Statistical analysis

The data were analyzed using one-way analysis of variance (ANOVA) test. To assess the significance of differences the Tukey *post-hoc* test was used. P values less than 0.05 were statistically significant. Graphs were performed using Prism software program (graphpad prism software, version 8.2, CA, USA) and analysis of data was performed using GraphPadInStat, Version 8.2.

## Results

### Effect of radiation and/or astaxanthin on the growth of human MCF7 cancer cells

**Fig. 1:**
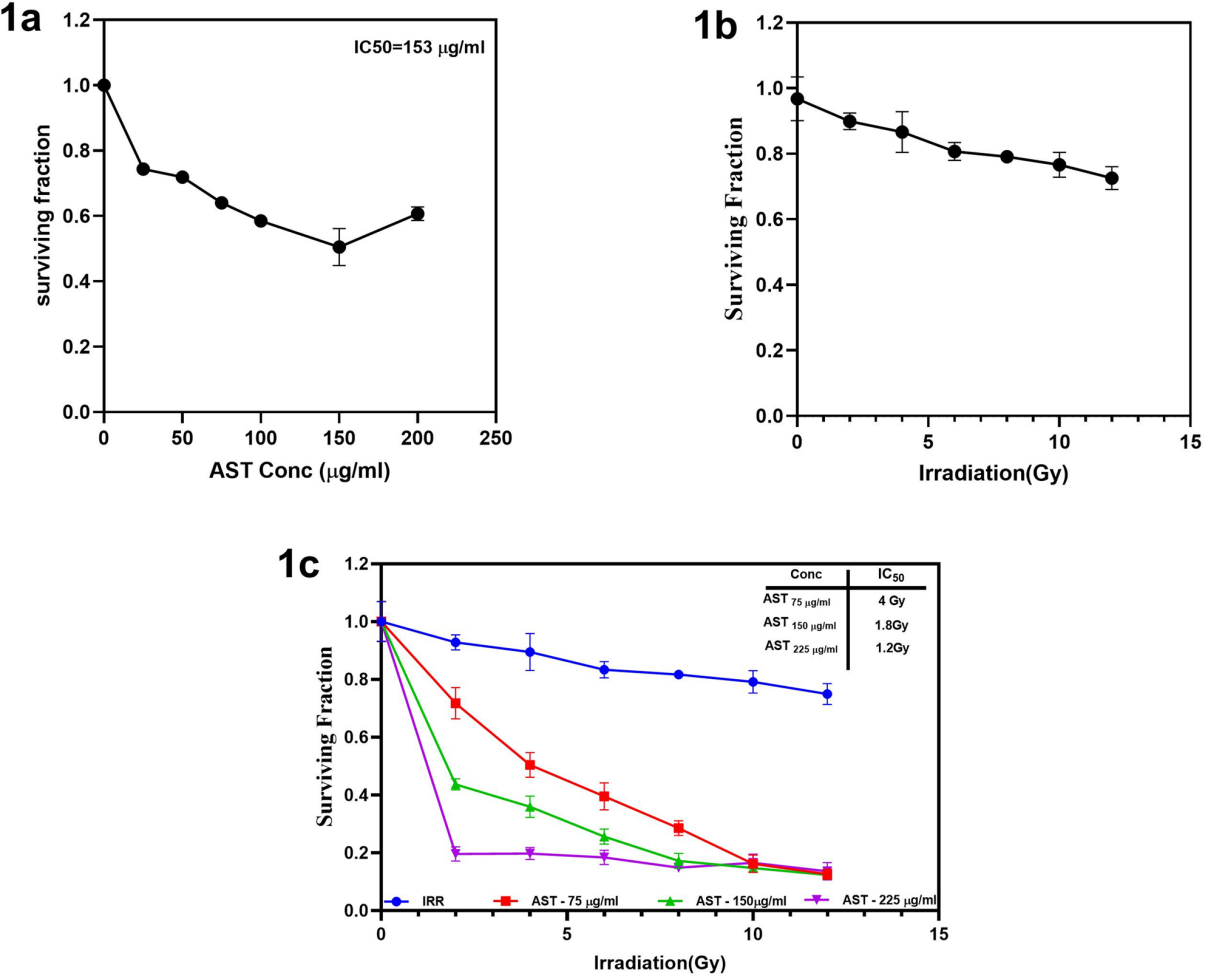
Cytotoxic effect of radiation and/or astaxanthin on human MCF7 cancer cells (**a**) AST alone, (**b**) IRR alone and (**c**) is IRR+AST after 48 hr incubation. The results are expressed as the mean ± SD of 2 separate experiments performed in 6 replicates

Figure (1a) shows that AST treatment alone exerted cytotoxic activity against the growth of MCF7 cells, with an IC_50_ of 153 µg/ml ± 0.05. On the other hand, exposure to radiation alone at doses (2, 4,6,8,10 and 12 Gy) did not exert a significant cytotoxic activity on MCF7 cells (Fig. 1b), where an IC_50_ could not be reached. However, in presence of AST, radiation showed a significant cytotoxic activity against the growth of MCF7 cells with IC_50_ of 4, 1.8 and 1.2 Gy at AST doses of 75, 150 and 225 µg/ml, respectively, (Fig. 1c).

### Effect of radiation and/or astaxanthin on antioxidant activities in MCF7 cells

**Fig. 2:**
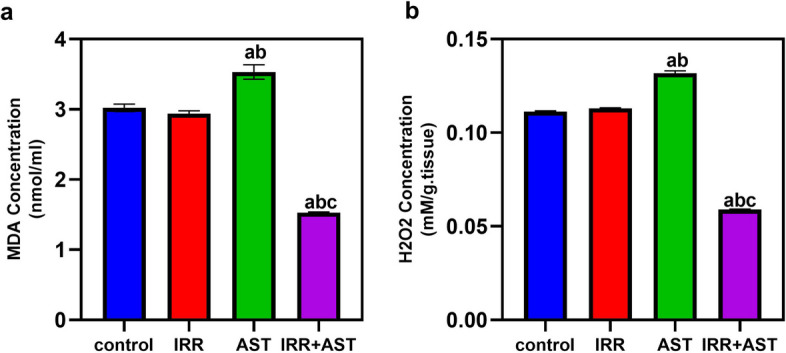
Effect of radiation (4 Gy) and/or astaxanthin (75μg/ml) on concentrations of MDA (**a**), and H_2_O_2_ (**b**) in MCF-7 cells after 48 h incubation. The results are the mean ± SD of 2 separate experiments performed in triplets. ^a, b^ and ^c^ indicate statistically significant difference from the control, IRR and AST groups, respectively, at (*P* < 0.05) using one-way analysis of variance (ANOVA) followed by Tukey as a post-hoc test

Exposure of MCF7 cells to AST (75μg/ml) for 48 h showed 16.9% & 18.9% increase in MDA and H_2_O_2_ concentrations, respectively compared to the control values. However, AST addition to IRR showed a significant decrease by 48% & 47.8% compared to IRR alone, respectively (Fig. 2a and b).

### Effect of radiation and/or astaxanthin on apoptotic activity in MCF7 cells

**Fig. 3:**
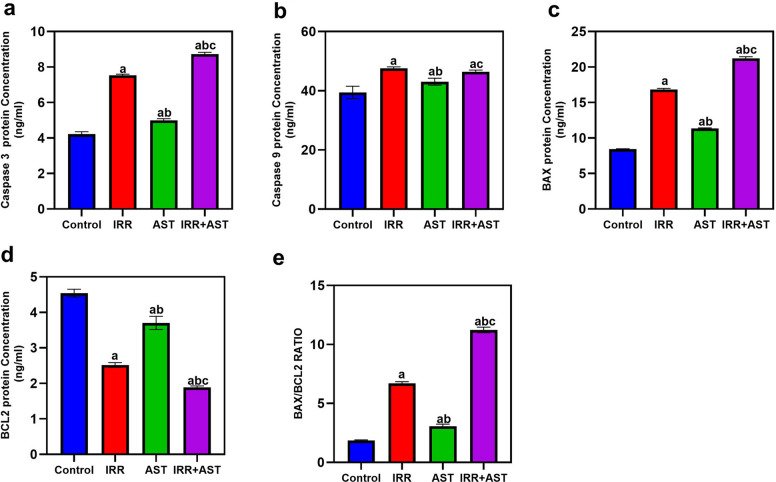
Effect of radiation (4 Gy) and/or astaxanthin (75μg/ml) on protein concentrations of caspase 3 (**a**), caspase 9 (**b**), BAX (**c**) & BCL-2 (**d**) and BAX/BCL-2 ratio (**e**) in MCF-7 cells after 48 h. incubation. The results are the mean ± SD of 4 separate experiments performed in triplets. ^a, b^ and ^c^ indicate statistically significant difference from the control, IRR and AST groups, respectively, at (*P* < 0.05) using one-way analysis of variance (ANOVA) followed by Tukey as a post-hoc test

Exposure of MCF7 cells to 4Gy IRR at rate of 0.588 cGy/sec. showed 78%, 20.5% & 100% increase in caspase-3, 9 and BAX protein concentrations, respectively as compared to the control values. Co-treatment of AST (75μg/ml) with IRR (4Gy) displayed more significant increases in caspase-3 and BAX protein concentrations by 15.9% & 26%, respectively compared to IRR alone, (Fig. 3a, b and c). However, the combination did not show significant difference in caspase-9 compared to IRR alone, (Fig. 3b). On the other hand, exposure of MCF7 cells to 4Gy IRR showed a significant decrease in BCL2 protein concentrations by 44.6% compared to control, while addition of AST showed a significant decrease by 24.9% compared to IRR alone (Fig. 3d). Moreover, the ratio of BAX/BCL2 increased after combination treatment by 67.8% compared to radiation alone (Fig. 3e).

### Effect of radiation and/or astaxanthin on metastatic activity in MCF7 cells

**Fig. 4:**
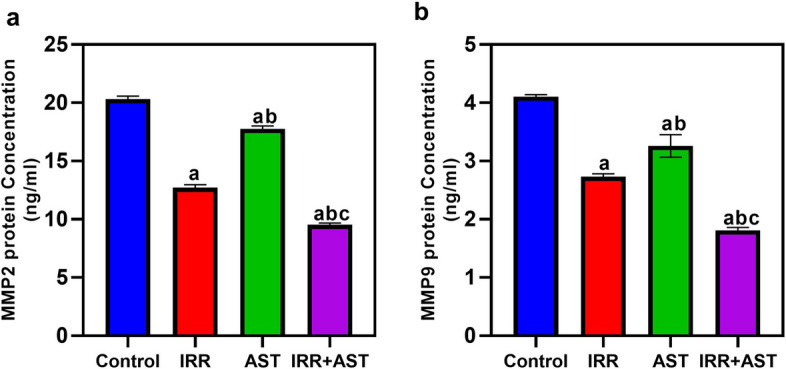
Effect of radiation (4 Gy) at rate of (o.588 cGy/sec.) and/or astaxanthin (75μg/ml) on protein concentrations of MMP2 (**a**) and MMP9 (**b**) in MCF-7 cells after 48 h. incubation. The results are the mean ± SD of 2 separate experiments performed in triplets. ^a, b^ and ^c^ indicate statistically significant difference from the control, IRR and AST groups, respectively, at (*P* < 0.05) using one-way analysis of variance (ANOVA) followed by Tukey as a post-hoc test

As compared to control, exposure of MCF7 cells to 4Gy IRR at rate of 0.588 cGy/sec showed 37.4% and 33.4% decrease in MMP2 and MMP9 protein concentrations, respectively. In the presence of AST, IRR displayed more significant decreases by 25% & 33.8%, respectively as compared to IRR alone (Fig. 4a and b).

### Effect of radiation and/or astaxanthin on DNA repair activity in MCF7 cells

**Fig. 5:**
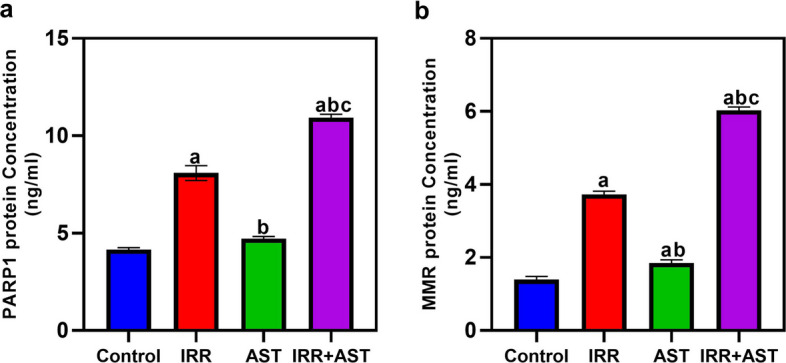
Effect of radiation (4 Gy) at rate of (o.588 cGy/sec.) and/or astaxanthin (75μg/ml) on protein concentrations of PARP-1 (**a**) and MMR (**b**) in MCF-7 cells after 48 h. incubation. The results are the mean ± SD of 2 separate experiments performed in triplets. ^a, b^ and ^c^ indicate statistically significant difference from the control, IRR and AST groups, respectively, at (*P* < 0.05) using one-way analysis of variance (ANOVA) followed by Tukey as a post-hoc test

Ionizing irradiation (4Gy) at rate of 0.588 cGy/sec. significantly increased the protein concentrations of PARP-1 and MMR by 94.7% & 167%, respectively compared to the control values. However, addition of AST showed significant increases by 35.2% & 61.7% in PARP-1 and MMR protein concentrations compared to IRR alone, respectively (Fig. 5a and b).

### Effect of radiation and/or astaxanthin on CDK4 and CDK6 protein concentrations in MCF7 cells

**Fig. 6:**
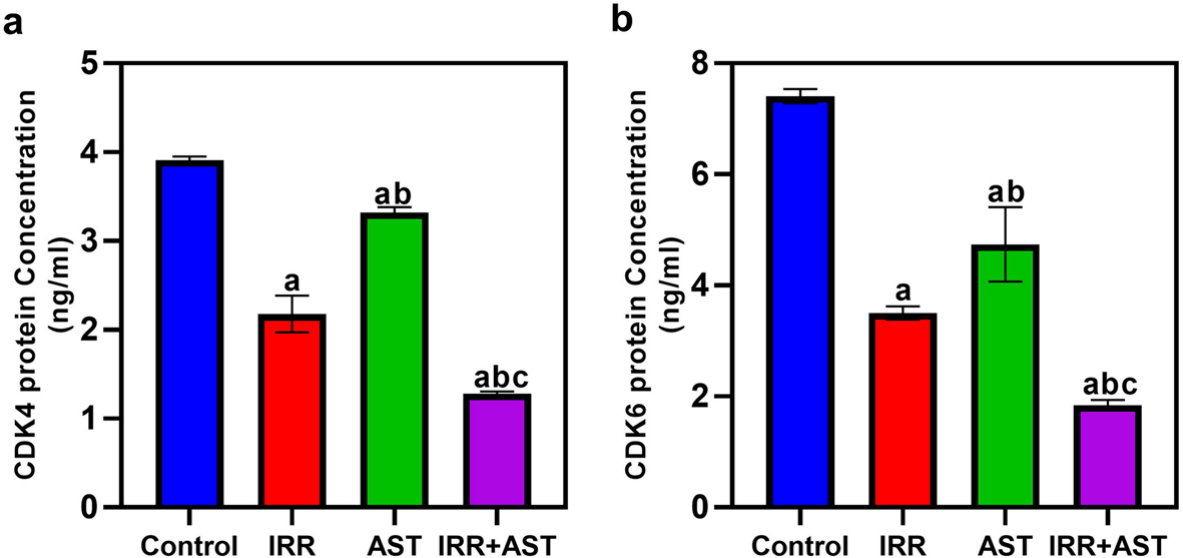
Effect of radiation (4 Gy) at rate of (o.588 cGy/sec.) and/or astaxanthin (75μg/ml) on protein concentrations of CDK4 (**a**) and CDK6 (**b**) in MCF-7 cells after 48 h. incubation. The results are the mean ± SD of 2 separate experiments performed in triplets. ^a, b^ and ^c^ indicate statistically significant difference from the control, IRR and AST groups, respectively, at (*P* < 0.05) using one-way analysis of variance (ANOVA) followed by Tukey as a post-hoc test

Upon exposure of MCF7 cells to 4Gy IRR at rate of 0.588 cGy/sec, CDK4 and CDK6 protein concentrations were significantly decreased by 44.3% & 52.7%, respectively, compared to the control values. Additional decreases by 41.2% & 47.4%, respectively compared to IRR alone were observed in the presence of AST (75μg/ml) (Fig. 6a and b).

### Effect of radiation and/or astaxanthin on cell cycle phase distribution in MCF7 cells

**Fig. 7:**
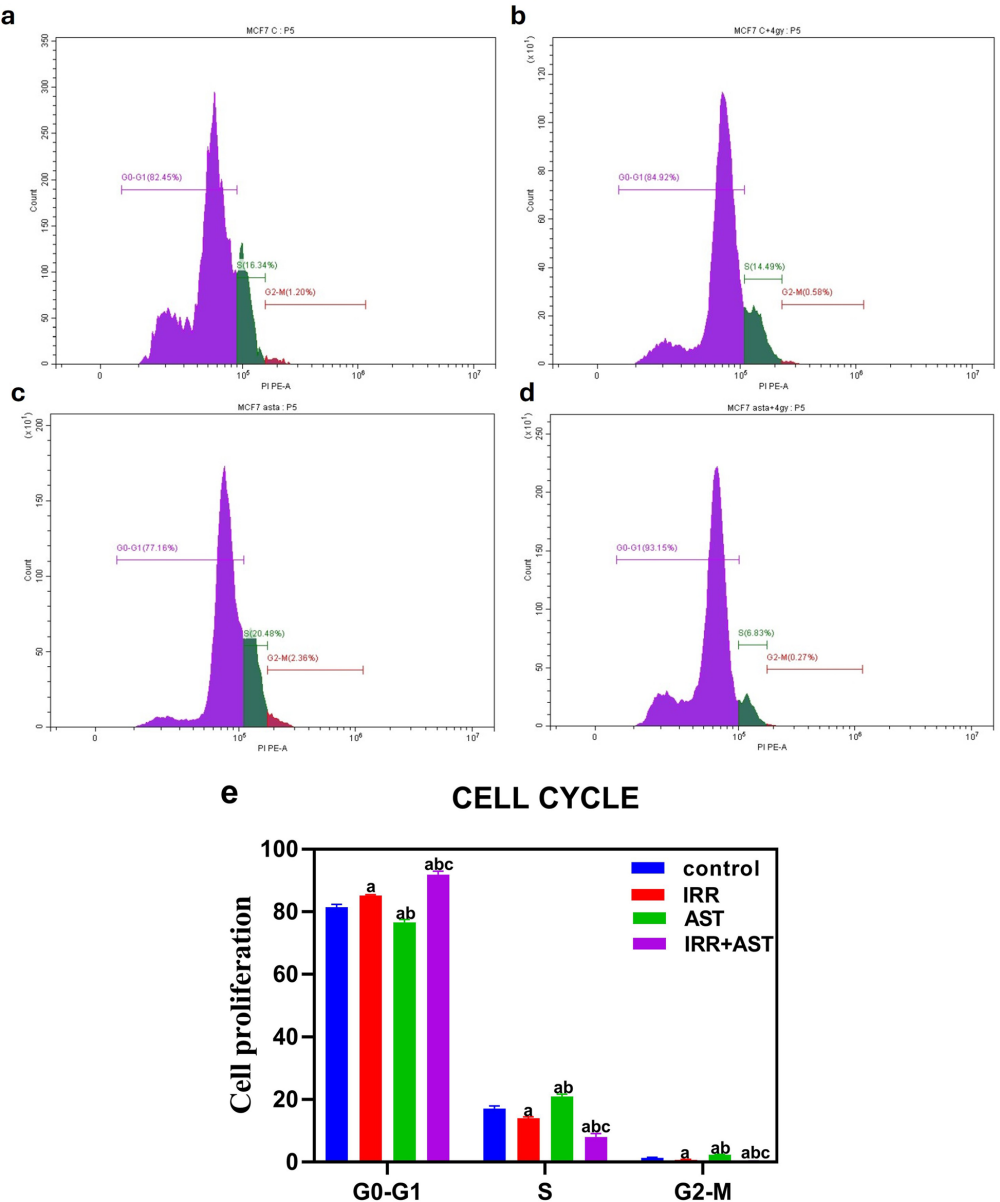
Effect of radiation (4Gy) at rate of (o.588 cGy/sec.) and/or astaxanthin (75μg/ml) after 48 h incubation on Cell Cycle Phase Distribution of MCF-7 cells. *a control, **b** IRR, **c** AST, **d** IRR+AST & **e** cell cycle histogram.* The results are the mean ± SD of 4 separate experiments performed in triplets. ^a, b^ and ^c^ indicate statistically significant difference from the control, IRR and AST groups, respectively, at (*P* < 0.05) using one-way analysis of variance (ANOVA) followed by Tukey as a post-hoc test

The exposure of MCF7 cells to combination of AST 75μg/ml and IRR 4Gy showed significant increase in the percentage of accumulation of cells in G_0_/G_1_ phase by 7.8%. In addition, significant decreases in G_2_/M and S phases by 52.1% and 42.6%, respectively compared to IRR alone (Fig. 7b, d and e).

### Effect of radiation and/or astaxanthin on proliferation indices (PI) and S phase ratio in MCF7 cells

In Table 1 the exposure of MCF7 cells to combination of IRR 4Gy and of AST 75μg/ml showed significant decrease in the proliferation index and S phase ratio of cells by 43.24% and 42.6%, respectively as compared to IRR alone. In contrast, AST alone significantly increased the proliferation index and S phase ratio of cells by 25.27% and 22.1% compared to control, respectively.

**Table 1:** Effect of radiation and/or astaxanthin on proliferation indices (PI) and S phase ratio in MCF7 cells

Treated groups	proliferation indices (PI) in MCF7 (%)	S phase ratio (%)
Control	18.6 ± 0.010	17.2 ± 0.008
IRR	14.8 ± 0.003^a^	14.1 ± 0.004^a^
AST	23.3 ± 0.008^a,b^	21 ± 0.007^a,b^
IRR + AST	8.4 ± 0.011^a,b,c^	8.1 ± 0.011^a,b,c^

Effect of radiation (4 Gy) at rate of (o.588 cGy/sec.) and/or astaxanthin (75μg/ml) after 48 h. incubation on proliferation indices (PI = G_2_/M + S phase %) and % of S phase ratio of MCF-7 cells. The results are the mean ± SD of 4 separate experiments performed in triplets. a, b and c indicate statistically significant difference from the control, IRR and AST groups, respectively, at (*P* < 0.05) using one-way analysis of variance (ANOVA) followed by Tukey as a post-hoc test

## Discussion

Radiotherapy (RT) is considered as a primary option for many types of cancer treatment either alone or in combination with surgery and chemotherapy [3]. Its effectiveness is restricted as it may cause serious side effects [3, 4]. Many strategies have been investigated to improve the therapeutic activity of chemo and radiotherapy so decreasing the dose and lower their toxicity [14–17]. Radiosensitization by natural product is one technique that have been utilized [14]. Therefore, in this study we investigated the modulatory role of natural product astaxanthin as radiosensitizer against the growth of MCF-7 cells. AST is proved as a food colorant and antioxidant [8, 18].

In the current study, exposure of MCF-7 cells to different doses of ionizing irradiation (IRR) did not show significant cytotoxic activity against the growth of MCF7 cells. While in presence of AST, cytotoxic activity was significantly observed (Fig. 1b) with IC50 decreased by about 10 folds. These results have been confirmed by induction of apoptosis presented by increasing protein concentrations of caspase 3, 9 and BAX as well as BAX/BCL2 ratio (Fig. 3a, c &e).

Evidence on the potential effect of AST as a radiosensitizer is still scarce [19]. In the present work, AST alone has a prooxidant effect on MCF-7 cells shown by increasing the concentrations of MDA and H_2_O_2_ (Fig. 2a & b). This is in agreement with Shin et al. who reported that carotenoids act as an antioxidant in normal cells, but they act as a pro-oxidant in cancer cells [20]. Several studies also reviewed that despite carotenoids having anti-oxidant effect, they act as a pro-oxidant under certain conditions. They also assumed that there are several factors in which carotenoid will act as anti-oxidant or pro-oxidant including, carotenoid concentration and the redox environment of the cell [21, 22]. It has been reported that increase in BAX/BCL2 ratio decrease the cellular resistance to apoptotic stimuli, leading to increase cell death and reduced incidence of tumor [23]. Cepero et al. showed that activation of caspase 9 increases ROS production by preventing cytochrome C accessibility to complex Ⅲ and that effector caspases (caspase 3) may depolarize mitochondria to inhibit ROS production and restore an apoptotic phenotype [24, 25]. The above-mentioned results agree with the current study where caspase 3 concentration significantly increased in combination (IRR + AST) therapy compared to IRR alone without significant change in caspase 9 concentration (Fig. 3a&b). This may explain the increase in the cytotoxic activity of combination therapy, while MDA and H_2_O_2_ concentrations (oxidative stress) have been decreased (Fig. 2a & b).

Addition of AST has been found to increase the antimetastatic activity of IRR by decreasing protein concentrations of MMP-2 and MMP-9 (Fig. 4a & b). This agrees with that reported by Gong et al. who highlighted the antimetastatic function of AST in clear cell renal carcinoma (CCRCC) suggesting its therapeutic potential in cancer metastasis alone and in combination with RT [26].

Ionizing irradiation (IRR) alone showed a significant increase in PARP-1 protein concentration, however addition of AST, induced more significant increase (Fig. 5a). This could be attributed as compensatory mechanism by the cells to correct the severe DNA damaging effect of the combination therapy, since PARP1is a primary signaling factor in DNA damage response [27], with release and trapping of PARP1 at sites of DNA damage [28].

It has been reported that cells deficient in MMR are relatively resistant to alkylation damage because MMR system is thought to promote toxicity via rutile repair of alkylated mispairs [29]. This is in line with the current study where AST increased cytotoxicity of IRR and at the same time increased the level of MMR activity, (Fig. 5a and b). Because IRR induce a variety of lesions in DNA, so the difference in survival between IRR alone and combination of IRR and AST may reflect a role for MMR in processing a subset of these lesions, such as damaged bases.

AST alone has a synchronized effect on the cells in S phase (Fig. 7b, d & e and Table 1) which is the most sensitive phase for IRR action [30]. Moreover, addition of AST to IRR showed a significant decrease in protein concentrations of CDK4 and CDK6 (Fig. 6a&b) causing cell cycle arrest and decrease in proliferation index and S phase ratio (Table 1). These results prove and confirm the more cytotoxic activity of RT in presence of AST against the growth of MCF-7 cells. However, Du et al. showed that AST might synergize IRR effect on oral squamous cell carcinoma (OSCC) both in vitro and in vivo, by other mechanisms partly via ferroptosis [31], that is a form of lipid peroxidation-induced nonapoptotic cell death.

## Conclusion

Taken together, all the above-mentioned results showed that AST may sensitized IRR by its apoptotic and antimetastatic effect against the growth of MCF7 cells. In addition, AST showed a synchronized effect at S, G2/M phase which is known to be more sensitive phase to the action of IRR.

## Data Availability

The article and its supplemental information contain all of the data created or analyzed during this study are available from the corresponding author on reasonable request.

## Abbreviations

RT: Radiotherapy
AST: Astaxanthin
IRR: Ionizing irradiation
MDA: Malondialdehyde
PI: Proliferation index
H_2_O_2_: Hydrogen peroxide
